# Coping during COVID-19: a mixed methods study of older cancer survivors

**DOI:** 10.1007/s00520-020-05929-5

**Published:** 2021-01-06

**Authors:** Jacqueline Galica, Ziwei Liu, Danielle Kain, Shaila Merchant, Christopher Booth, Rachel Koven, Michael Brundage, Kristen R. Haase

**Affiliations:** 1 School of Nursing, Queen’s University, Kingston, Canada; 2 Division of Cancer Care & Epidemiology, Queen’s Cancer Research Institute, Kingston, Canada; 3 Division of Palliative Medicine, Departments of Medicine and Oncology, School of Medicine, Queen’s University, Kingston, Canada; 4 Division of General Surgery and Surgical Oncology, Queen’s University, Kingston, Canada; 5 Cancer Centre of Southeastern Ontario, Kingston, Canada; 6 Department of Oncology, School of Medicine, Queen’s University, Kingston, Canada; 7 Department of Public Health Sciences, School of Medicine, Queen’s University, Kingston, Canada; 8 School of Nursing, Faculty of Applied Science, The University of British Columbia, Vancouver, Canada

**Keywords:** Cancer, Mixed methods, Older adults, Qualitative, Coping skills, Coping behaviors

## Abstract

**Purpose:**

Older cancer survivors are among the most vulnerable to the negative effects of COVID-19 and may need specific survivorship supports that are unavailable/restricted during the pandemic. The objective of this study was to explore how older adults (≥ 60 years) who were recently (≤ 12 months) discharged from the care of their cancer team were coping during the pandemic.

**Methods:**

We used a convergent mixed method design (QUAL+quan). Quantitative data were collected using the Brief-COPE questionnaire. Qualitative data were collected using telephone interviews to explore experiences and strategies for coping with cancer-related concerns.

**Results:**

The mean sample age (*n* = 30) was 72.1 years (SD 5.8, range 63–83) of whom 57% identified as female. Participants’ Brief-COPE responses indicated that they commonly used acceptance (*n* = 29, 96.7%), self-distraction (*n* = 28, 93.3%), and taking action (*n* = 28, 93.3%) coping strategies. Through our descriptive thematic analysis, we identified three themes: (1) drawing on lived experiences, (2) redeploying coping strategies, and (3) complications of cancer survivorship in a pandemic. Participants’ coping strategies were rooted in experiences with cancer, other illnesses, life, and work. Using these strategies during the pandemic was not new—they were redeployed and repurposed—although using them during the pandemic was sometimes complicated. These data were converged to maximize interpretation of the findings.

**Conclusions:**

Study findings may inform the development or enhancement of cancer and non-cancer resources to support coping, particularly using remote delivery methods within and beyond the pandemic. Clinicians can engage a strengths-based approach to support older cancer survivors as they draw from their experiences, which contain a repository of potential coping skills.

## Introduction

In 2020, severe acute respiratory syndrome coronavirus 2 (SARS-CoV-2, herein called COVID-19) affected numerous people around the world [1]. Those most vulnerable to the negative effects of COVID-19 include those with comorbid conditions [2] (e.g., cancer [3]) and those over age 60 [3] to 65 years [2]. Higher fatality rate and complications of COVID-19 are associated with advanced age and presence of chronic diseases [4]. Given that nearly 9 out of 10 cancer diagnoses occur among people 60 years of age and older [5], older adults living with cancer are an important group to study during this pandemic.

Since the beginning of the COVID-19 pandemic, reports have detailed additional psychological distress associated with being a cancer survivor [6]. Cancer survivors recently discharged from the care of their cancer team and no longer receiving cancer treatment cite a number of unmet needs after completing cancer treatment [7–9] (e.g., for psychosocial and informational support [7–10]) and describe the first 6–12 months after treatment as the most difficult [7]. Prior to the pandemic, most supports and resources available to cancer survivors recently (≤ 12 months) discharged from the care of their cancer team were provided as face-to-face appointments with professionals [4]; however, in adherence to COVID-19’s requirement for physical distancing [1], many in-person cancer-related resources are no longer available to survivors leaving them to use alternative means for coping, methods which may be untested and potentially more debilitating to long-term psychosocial well-being [11]. Considering that older adults are among the most vulnerable groups [2, 3], and that the COVID-19 pandemic could further heighten the psychological distress they experience as a cancer survivor [6], suggest that older adult cancer survivors require additional consideration.

A variety of theoretical explanations and operationalizations of coping exist. Moos and Holahan [12] proposed a coping framework describing the interconnections among characteristics of how individuals cope. Personal resources [12] include characteristics of the individual, such as dispositional coping style and characteristics such as cognitive and intellectual ability and general personality traits [12]. The environmental system encompasses relatively stable characteristics, such as ongoing life stressors and social resources (e.g., physical health, finances, relationships) [12]. These systems influence a cascade of processes that affect the individual’s psychosocial functioning and well-being. Moos and Holahan [12] describe coping in two domains: coping styles and coping responses. Coping styles are relatively stable and describe how individuals habitually interact with their environment, whereas coping responses are used by the individual to manage a specific stressful encounter [12]. This framework may inform how older cancer survivors may cope with the experience of two simultaneous but distinct events: a recent discharge from the cancer center and the COVID-19 pandemic.

Although prominent national [13, 14] and international [15, 16] cancer organizations have availed resources to inform cancer patients and survivors about their susceptibility to the negative physiological effects of COVID-19, no known studies have assessed the psychosocial implications of COVID-19 on post-treatment of older cancer survivors.

### Purpose and research questions

The purpose of this manuscript is to understand coping among older cancer survivors (≥ 60 years of age) recently (≤ 12 months) discharged from a regional cancer center. The specific research questions are:

1. How does the COVID-19 pandemic affect access to formerly used and/or desired resources/supports?
2. What coping resources are older cancer survivors using during the pandemic?
3. How do these resources help (or not) to meet their cancer-related care needs?

## Methods

We used a convergent mixed methods (QUAL+quan) design—where quantitative and qualitative data were collected in parallel, analyzed separately, and then integrated to maximize interpretation of the findings [17].

### Sample and sampling

**Fig. 1:**
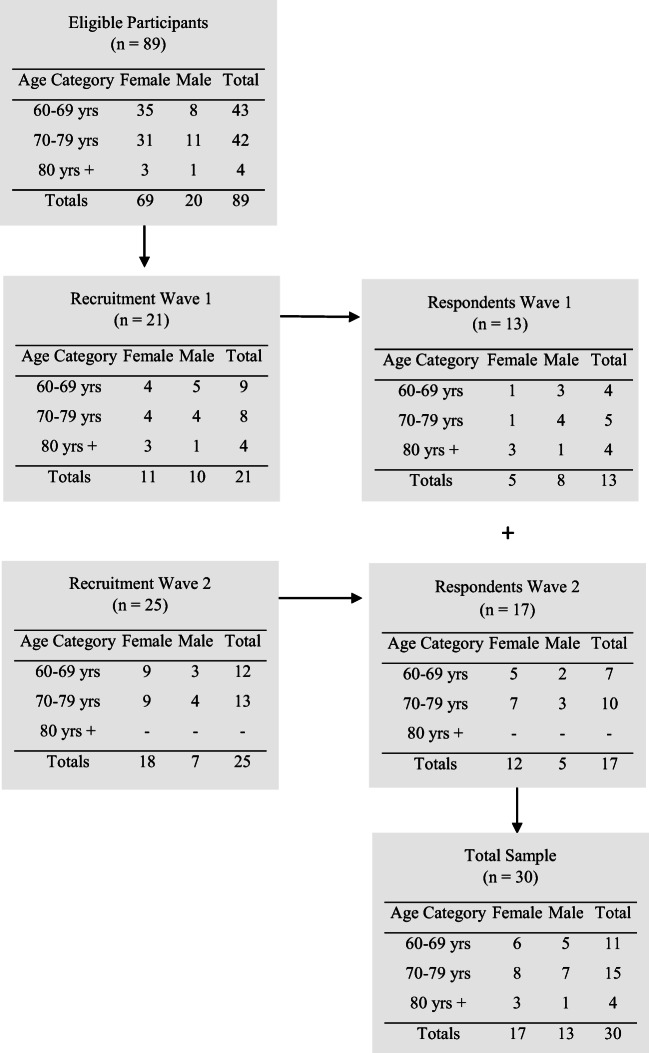
Recruitment strategy

Potential participants were drawn from a database of persons who had (1) been discharged from the care of their cancer team at the Cancer Centre of Southeastern Ontario in Kingston, Ontario, Canada, and (2) were agreeable to be contacted for further research conducted by the first author (JG). We used a stratified sampling method [18] to promote sample diversity among those at greatest risk for COVID-19 [2, 3] (e.g., age categories 60–69; 70–79, and ≥ 80 years) and mailed a consent form, the Brief-COPE [19] (described below), and a return-addressed postage-paid envelope to potential participants in 2 waves of recruitment (see Fig. 1). Interested persons returned a signed consent form and completed the Brief-COPE [19] (described below) to the first author (JG), whom they also contacted to make mutually convenient arrangements to complete a telephone interview. Reminder letters were mailed to participants who returned a signed consent form and completed Brief-COPE but who had not yet called to schedule a telephone interview. Reasons for not participating were not collected. All participants consented to have their self-reported demographic and clinical information (e.g., age, marital status, work status, ethnicity, diagnosis, and treatment) contained in the first author’s database to be used in the current study. Ethical review of the study was obtained prior to beginning the study (Queen’s University HSREB#6030148).

### Sample size

In alignment with qualitative methods, sample size is a matter of both feasibility, judgment, and expertise [20]. In this study, we sought to ensure diverse and robust perspectives among recruited participants and interviewed until we noted repetition in themes, and a detailed description of the phenomena was developed based on participant accounts [21].

### Data collection

Experienced qualitative researchers (JG, KH) collected data via 1:1 interviews that were guided by a semi-structured interview guide (see Table 1 for examples). The telephone interviews lasted between 25 and 60 min, were digitally recorded, and professionally transcribed.

**Table 1:** Sample interview questions asked of study participants

• *Prior* to the COVID-19 pandemic, could you tell me some of the *individual* things that you did to cope with your cancer-related concerns and how these worked for you? • *Prior* to the COVID-19 pandemic, could you tell me some of the *more formal or organized resources* you used to cope with your cancer-related concerns and how these worked for you? • Could you tell me some of the *individual* things that you are doing to cope with your cancer-related concerns *during* COVID-19 and how are these working for you. Please explain. • Could you tell me about *more formal or organized resources* that you are using for coping with your cancer-related concerns *during* COVID-19 and how these are working for you? • What do you believe are some of your personal characteristics or attributes that help you cope with your cancer-related concerns *during* the pandemic? • Of all the changes that you have gone through as an older cancer survivor during this pandemic, what stands out to you as the most important change you have had to make to cope with your cancer-related concerns?	

Quantitative data was collected using the Brief-COPE [19], a 28-item measure assessing 14 coping responses: active coping, planning, positive reframing, acceptance, humor, religion, using emotional support, using instrumental support, self-distraction, denial, venting, substance use, behavioral disengagement, and self-blame [19]. On the Brief-COPE, participants indicate ways that they cope with any stress in their life and use a 4-point Likert scale to respond (1 = I do not do this at all, to 4 = I do this a lot). Although the Brief-COPE does not provide an overall coping score [22], a three factor model^1^ of coping has been supported in a sample of breast cancer patients [23] and reflects the framework of coping [12] used to guide this study: For instance, the self-sufficient and avoidant coping factors [23] were deemed to reflect strategies involving Moos and Holahan’s (2003) personal system, whereas the socially supported coping factor [23] was deemed to reflect strategies involving the environmental system. The psychometric properties of the Brief-COPE have been evaluated in samples of cancer survivors [24, 25]. The internal consistencies of subscales in the current sample are indicated in Table 2.

**Table 3:** Demographic and clinical characteristics of the sample

Demographic characteristics	*N* (%)	Clinical characteristics	*N* (%)
Age (years)^A^	72.1 (5.8)	Diagnosis	
Gender		Breast cancer	15 (50.0)
Male	13 (43.3)	Colon or rectal cancer	15 (50.0)
Female	17 (56.7)	Received chemotherapy^B^	
Marital status		Yes	29 (96.7)
Married or common-law	23 (76.7)	No	0 (0)
Widowed	3 (10.0)	Time (months) since chemotherapy^A^	20.0 (13.4)
Separated or divorced	1 (3.3)	Received radiation therapy^B^	
Single (never married)	3 (10.0)	Yes	17 (56.7)
Parental status		No	12 (40.0)
Have children	23 (76.7)	Time (months) since radiation therapy^A^	24.6 (12.8)
No children	7 (23.3)	Received other cancer treatment^B,C^	
Education		Yes	9 (30.0)
Up to high school graduate	9 (30.0)	No	20 (66.7)
Up to post-secondary graduate	14 (46.7)	Time (months) since other treatment^A^	24.2 (13.5)
Up to graduate-level graduate	7 (23.3)	Medication use^B^	
Current work status		Not taking medication	5 (16.7)
Retired/not working	28 (93.3)	For cancer only	6 (20.0)
Working at a job/business	2 (6.7)	For cancer and non-cancer	2 (6.7)
Resides in		For non-cancer only	16 (53.3)
Urban location	19 (63.3)		
Rural location	11 (36.7)		
Ethnicity			
Caucasian	28 (93.3)		
Non-Caucasian	2 (6.7)		
Religious or spiritual			
Yes	16 (53.3)		
No	14 (46.7)		

^A^Mean (SD)

^B^Missing (*n* = 1)

^C^As indicated by participants (e.g., immunotherapy, surgery, injections)

### Data analysis

We used a descriptive thematic analytic approach [26, 27], wherein the goal was to summarize and describe patterns and meaning in the data to understand the context and nuance around older cancer survivors’ experiences of coping with cancer-related concerns during the pandemic. Two team members (JG, KH) engaged in the qualitative data analysis using NVivo 12, with weekly meetings to discuss the developing coding framework and impressions of the data. Transcripts were read and re-read to assign initial codes, organized into sub-themes and broader themes, until consensus was reached to provide an inclusive thematic description.

Quantitative data was analyzed using SPSS version 26. Descriptive statistics for participant demographics and Brief-COPE [19] data were calculated and used to provide context to the qualitative results related to the study objectives.

## Results

### Quantitative

The sample was, on average, 72.1 years of age (SD 5.8, range 63–83 years) and comprised 17 female and 13 male participants. Most participants identified as Caucasian (93%), were married or common-law (76%), and retired or not working outside the home (93%). Equal numbers of the sample had a breast or colorectal cancer diagnosis, and most had received chemotherapy (96%) that was completed an average of 20 months previously (SD 13.4, range 0–52 months). Full details are presented in Table 3.

**Table 2:** Brief-COPE results

	Brief-COPE factors, subscales^a^, and items	Cronbach’s alpha	*N* (%) using the coping strategy
Personal system	Self-sufficient coping factor [23]		
	Acceptance subscale	0.50	29 (96.7)
	20. Accept that it has happened		28 (93.3)
	24. Learn to live with it		29 (96.7)
	Active subscale	0.68	28 (93.3)
	2. Doing something about the situation I’m in		26 (86.7)
	7. Try to make situation better		25 (83.3)
	Planning subscale	0.65	27 (90.0)
	14. Come up with strategy about what to do		25 (83.3)
	25. Think hard about what steps to take		27 (90.0)
	Positive reframing subscale	0.66	26 (86.7)
	12. Try to see it in a different light		25 (83.3)
	17. Look for something good		25 (83.3)
	Humor subscale	0.81	19 (63.3)
	18. Make jokes about it		18 (60.0)
	28. Make fun of the situation		16 (53.3)
	Avoidant coping factor [23]		
	Self-distraction subscale	0.72	28 (93.3)
	1.Turn to work or other activities as distraction		27 (90.0)
	19. Do something to think about it less		26 (86.7)
	Self-Blame Subscale	0.77	19 (63.3)
	13. Criticize myself		19 (63.3)
	26. Blame myself for things that happened		7 (23.3)
	Behavioral disengagement subscale	0.71	10 (33.3)
	6. Give up trying to deal with it		7 (23.3)
	16. Give up the attempt to cope		6 (20.0)
	Denial subscale	0.57	8 (26.7)
	3. Say to myself “this is not real”		5 (16.7)
	8. Refuse to believe it happened		6 (20.0)
Environmental system	Socially supported coping factor [23]		
	Instrumental support subscale	0.64	26 (86.7)
	10. Get help and advice from others		24 (80.0)
	23. Get advice or help about what to do		20 (66.7)
	Emotional support subscale	0.79	25 (83.3)
	5. Get emotional support from others		24 (80.0)
	15. Comfort and understanding from someone		20 (66.7)
	Venting subscale	0.68	23 (76.7)
	9. Say thing to let unpleasant feelings escape		17 (56.7)
	21. Express negative feelings		22 (73.3)
	Religion subscale	0.85	15 (50.0)
	22. Find comfort in religion or spiritual belief		13 (43.3)
	27. Pray or meditate		15 (50.0)

After dichotomizing item responses into “I do not do this at all” and all others, the *N* (%) for each item and subscale were determined

^a^The *N* (%) for the substance use subscale (items 4 and 11) were 3 (10%) and 2 (6.7%), respectively. The Cronbach’s alpha for the substance use subscale was 0.88

Most participants used coping strategies from their personal system to cope with stress in their life, such as taking actions to engage in their situation (*n* = 28, 93.3%), distract themselves from it (*n* = 28, 93.3%), or to accept it (*n* = 29, 96.7%). Environmental coping strategies were used less often, but of these strategies, many participants used instrumental (*n* = 26, 86.7%) or emotional support (*n* = 25, 83.3%) to cope with stress in their life.

### Qualitative

**Fig. 2:**
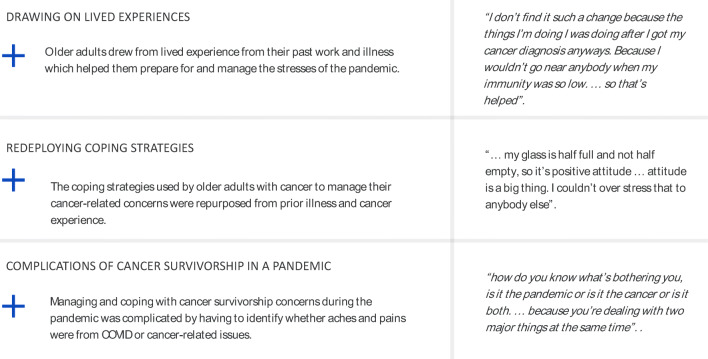
Exemplar quotes illustrating themes of coping experiences of older cancer survivors in the pandemic

We identified three interconnected themes to describe how older adult cancer survivors coped with their cancer-related concerns during the pandemic: (1) drawing on lived experiences, (2) redeploying coping strategies, and (3) complications of cancer survivorship in a pandemic (see Fig. 2).

#### Drawing on lived experiences

The multifaceted experiences that older cancer survivors learned through life, work, cancer, or other illness formed the basis of their coping strategies. Participants most commonly described how their cancer and non-cancer illness experiences (or the illness experiences of those close to them) informed their ability to respond to the pandemic. Some participants described how they had enacted coping strategies during their former illness experiences that they continued to use during the pandemic. In this way, although cancer survivors are seen as doubly vulnerable, they focused on the strengths and coping capacity they had gained from their prior illness experience.

Participants cited various life experiences that informed their coping strategies at this stage of life, of which the aging process was the most cited. One participant stated: “*I’m 73 years old, I’m in* *that decade where a lot of things can go wrong. … I’ve had* *a good life and I’m not prepared to die, but I’m not afraid of dying … if I had to go tomorrow, I wouldn’t think I’d have a lot of regrets*.” Participants associated aging with an increased likelihood of comorbid conditions, cancer, death, and COVID-19, which left them appreciating their life lived, and with few reservations about succumbing to COVID-19, despite having had cancer.

Participants appreciated how the privileges of financial planning for retirement, downsizing/renovating their living space, preparing living wills/funeral, and their living circumstances (e.g., not in long term care, in a rural area, an independent retirement community) provided ‘insulation’ from the negative effects of COVID-19. However, for one disabled participant living in a rural area, her in-home supportive services ceased due to the pandemic, which she said: “*made me very confined because everything in this country is based on driving a car. … I have to arrange for somebody to do the groceries … it has affected me in a big way*.”

Work experience—specifically healthcare-related fields—influenced how older cancer survivors coped with their cancer-related concerns. One participant was a healthcare professional who provided care in northern communities. She drew upon these experiences to cope during the pandemic: she said “*I have lived in isolation … I had all these supplies in that you would use for being isolated. I did it as a routine*.” Non-healthcare professionals also cited work-related skills that were useful to inform their coping. For instance, working as an engineer who appreciates “*the science behind things*” or having worked with computers and various software systems during their past careers illuminated work-related attributes that participants drew upon to support their coping.

#### Redeploying coping strategies

The strategies older cancer survivors used to cope during the pandemic were not new; they were redeployed and repurposed. One participant put it plainly stating: “*absolutely everything is easier post-cancer and chemo*.” Some tried to continue on as before the pandemic (e.g., dressing nice and doing hair; ensuring cancer follow-up screening, continuing on as normal) or even magnify their formerly used strategies during the pandemic.

Participants cited a variety of activities (see Table 4) employed to cope with cancer-related concerns during the pandemic. These activities kept participants distracted from the pandemic and their cancer-related concerns, as indicated by one participant who crocheted dishcloths and hand towels: “*that’s like a therapy to me because it keeps my hands busy. It keeps my mind going. And it keeps my mind off of other things*.” In some instances, older cancer survivors stated that they did not have any cancer-related concerns, avoided thinking about their cancer-related concerns or needed to redirect their negative thoughts through self-talk: “*get it out of your head right now because you gotta live in the moment*.” Still some participants were more passive in their coping whereby they lived with their cancer-related concerns or chose not to take “*anything on right now*” until some normalcy returned into their life.

**Table 4:** Activities older cancer survivors used to stay busy/active during the COVID-19 pandemic

• Garden/yard work, farming	• Meditation/deep breathing
• Reading	• Spending time on computer
• Golf	• Going for walks
• Watching TV	• Fitness regime
• Doing puzzles	• Tai chi/yoga
• Knitting/crocheting, sewing	• Volunteering
• Restoring vehicles	• Fishing
• Coloring, painting	• Carpentry
• Massage therapy	• Playing an instrument
• Faith-based activities	

To make sense of the pandemic and how it influenced their cancer-related concerns, participants spoke of the importance of asking questions and documenting their condition daily so as to promote their sense of comfort and understanding about their health. Participants also recognized the challenges of receiving answers to their cancer-related concerns from healthcare professionals during the pandemic. To address their concerns during the pandemic, some participants used the Internet or literature that they already had, although they appreciated the importance of accessing information from trusted organizations identified by their healthcare providers. Participants felt that this information was useful to make decisions about whether to call their healthcare providers for more guidance or information and what questions to ask if they did so.

Maintaining social support during the pandemic was a key concern for participants. The Internet and technology-enabled interaction were seen as a lifeline to maintain connections (e.g., Skype, Facetime) with family and friends, but as the novelty wore off, these modalities became tiring. Apart from using technology, participants appreciated physically distant interactions with others, such as in hallways of their multi-unit residences, at the end of driveways, or in yards. One participant even went to a friend’s home for physically distanced dinner: “*She had extra leaves she put in the table - it was taking up the whole room - she and her husband were at one end and my husband and I were at the other*.” Undoubtedly, social support networks were pivotal to support older cancer survivors to cope with their cancer-related concerns as indicated by one participant: “*this pandemic hasn’t been that hard for me as compared to others that perhaps had no one to turn to and close friends or neighbours to rely on*.”

Some participants described a temporal aspect to coping strategies that had evolved over the course of the pandemic. While not everyone reported experiencing cancer-related concerns, some participants thought it was important to get on with life and even try new things. Some participants appreciated the new skills that they had been forced to learn during the pandemic, including ordering groceries online and using curb-side pick-up to comply with physical distancing recommendations. Others felt a loss of engagement in formerly enjoyed activities (e.g., not going to meet someone for coffee or leisurely shopping). Still other participants appreciated the opportunity to spend more time at home, while another felt the loss of “*freedom*” that the pandemic imposed.

#### Complications of cancer survivorship in a pandemic

Being newly discharged from cancer care, participants felt that they were coping well during the pandemic; however, upon further questioning, participants shared concerns about recurrence, social isolation, and missed opportunities to travel and see family. Many participants described the pandemic in the context of, or related to, their cancer, citing that they had survived cancer; therefore, the pandemic was just one more hurdle they needed to master or manage. They drew upon their lived experiences, and their many learned coping strategies to inform how they would get through the pandemic.

Many participants commented on the messy intersection of being an older cancer survivor and living in the pandemic. This was particularly true for participants who continued to experience side effects from cancer or other illnesses, which were often difficult to disentangle from each other, the aging process, or the possibility of COVID-19. These side effects worried participants because they did not know how long they would last, or they struggled to get resources to cope with these side effects during the pandemic. Lingering symptoms hindered participants’ ability to make health-related decisions or willingness to engage in formerly enjoyed activities, which sometimes led them to resign from the activity altogether. One participant stated: “*I have a breathing problem, so I really can’t just go to the store to shop because of the mask that I wear. It cuts my breathing way down*.”

On the contrary, some participants continued with their pre-pandemic activities but found that engaging in them was different than before. One participant described using fishing as a coping mechanism during cancer treatment but noted important differences now. He stated: “*I’ve been going fishing by myself rather than taking somebody with me, but it changes the way the fishing is. It’s just, there’s nobody to talk to or just shoot the breeze when you’re fishing*.” This notion was also applied to the use of technology to support social connections, which, as mentioned previously, was found to be tiring and less helpful over time because of the lack of direct personal interaction. Still other participants found this type of technology challenging and so preferred to use email or telephone to maintain contact with their friends and loved ones. Thus, the pandemic forced these older cancer survivors to adapt the coping mechanisms they had relied upon during their cancer diagnosis, which at times created frustration.

### Mixed methods comparison

Through triangulation of quantitative and qualitative data, we noted critical points of convergence and divergence. Overall, participants’ qualitative descriptions reflected their use of coping resources from their personal system, which aligned with the strategies most commonly identified on the quantitative measure. For instance, participants cited their own lived experiences (e.g., theme 1) as a foundation for their coping and identified activities (e.g., Table 4) that were, or could be, carried out on an individual basis. These activities reflect personal strategies on the quantitative measure, such as active coping with, or self-distraction from cancer-related concerns, which were among the most commonly used. Notably, an area of data divergence was found between the large proportions of participants who identified using venting or self-blame coping strategies on the quantitative measure (*n* = 23, 76.7% and *n* = 19, 63.3%, respectively), whereby these strategies were not described in the qualitative data.

## Discussion

In this mixed-methods inquiry, we sought to understand how older survivors of cancer were coping during the COVID-19 pandemic. We found that participants were most likely to use acceptance, self-distraction, and taking action to cope with their cancer-related concerns, coping strategies that stemmed from their personal system. We noted that participants drew on their lived experience, redeployed coping strategies and skills, and sought to understand how best to tackle the complications of transitioning to cancer survivorship during a pandemic. Because older adults have become one of the groups for whom the pandemic is thought to have negative health and social impacts [2, 28–30], this study lends insight into their experiences and provides initial ideas about how best to support older cancer survivors in this unique context.

We noted older cancer survivors’ exemplary resilience and numerous strengths as they described how they engaged their coping strategies from work, life, and past illness. While much research on older adults focuses on a deficit-based approach, we note how striking it is to see the considerable strengths of this population [31]. However, these participants noted many deficits in the services available to support them, which speaks to structural system-level challenges as opposed to individual vulnerabilities. This is especially important to note in the context of participants’ comments about privileged retirements and how those with less access to retirement funds and supports may bear an inordinate burden and be less equipped to cope with their cancer-related concerns during the pandemic. The amplification of ageism during the pandemic has been noted elsewhere [32, 33], but these findings serve as an additional reminder that structural resources tailored towards older adults' existing strengths may enhance the cancer survivorship transition for older adults.

While older adults expressed increasing comfort with technology-based resources [29, 34], participants in this study demonstrated an uneasiness and hesitancy around the absence of in-person clinic visits. Furthermore, while older adults found online platforms like Facetime and Zoom useful for social connection, they found it tiring over time and missed in-person interactions. Given the expected duration of the pandemic and the potential for other pandemics occurring in the future, there is a need to consider how best to support older adults to engage in activities that mitigate social isolation and promote physical health and well-being. Recent efforts to co-design resources with older adult cancer survivors may begin to address some of the present inadequacies [35].

### Strengths and limitations

The diverse ages of participants, the inclusion of persons in rural areas, and the near equal representation of men and women are key strengths of this study. Furthermore, the focus of this study reflects a current issue and adds to a small but expanding literature base about experiences of older cancer survivors. However, there are also limitations to this study, for example, we did not measure frailty or functional status, which are important considerations when studying older adults with cancer [36, 37]. Participants were recruited during the summer when social isolation and challenges related to physical distancing are potentially less concerning than in winter; therefore, these perspectives are representative of the participants’ views at the time during which the research was conducted. Lastly, while the sample is not meant to be representative, participants had been diagnosed with breast or colorectal cancer and were predominantly Caucasian and well-educated; therefore, results should be interpreted cautiously.

### Implications and conclusion

Study findings illuminate the strengths of older cancer survivors and their notable ability to adapt to pandemic-imposed limitations. These findings, in addition to the International Society of Geriatric Oncology (SIOG) recommendations [16], can be used to inform organizational responses to care during this and future pandemics. More specifically, study findings point to the importance of fostering strengths-based approaches where older adults can draw upon their personal systems for coping. As an initial step to support older survivors of cancer in this regard, tools could be developed to assist them to inventory their available coping resources. Study results can also inform the development of resources beyond the personal system that aim to support the self-management of physical and/or psychosocial well-being of older adults using remote delivery methods (e.g., telephone or online conferencing platforms). Indeed, research has begun to explore these topics, and future research could expand their implementation.

## Data Availability

Not applicable

## Footnotes

1 Due to its very low variance, the substance use subscale was not included in Wang and colleagues’ (2016) analysis [23].
